# Phytochemicals from *Polyalthia* Species: Potential and Implication on Anti-Oxidant, Anti-Inflammatory, Anti-Cancer, and Chemoprevention Activities

**DOI:** 10.3390/molecules26175369

**Published:** 2021-09-03

**Authors:** Yung-Chia Chen, Yi-Chen Chia, Bu-Miin Huang

**Affiliations:** 1 Department of Anatomy, School of Medicine, College of Medicine, Kaohsiung Medical University, Kaohsiung 80708, Taiwan; yungchia@kmu.edu.tw; 2 Graduate Institute of Medicine, College of Medicine, Kaohsiung Medical University, Kaohsiung 80708, Taiwan; 3 Department of Food Science and Technology, TaJen University, Pingtung 90741, Taiwan; ycchia@tajen.edu.tw; 4 Department of Cell Biology and Anatomy, College of Medicine, National Cheng Kung University, Tainan 70101, Taiwan; 5 Department of Medical Research, China Medical University Hospital, China Medical University, Taichung 40402, Taiwan

**Keywords:** *Polyalthia*, phytochemical, chemoprevention, anti-tumor, anti-cancer, anti-microbial

## Abstract

*Polyalthia* belong to the Annonaceae family and are a type of evergreen tree distributed across many tropical and subtropical regions. *Polyalthia* species have been used long term as indigenous medicine to treat certain diseases, including fever, diabetes, infection, digestive disease, etc. Recent studies have demonstrated that not only crude extracts but also the isolated pure compounds exhibit various pharmacological activities, such as anti-oxidant, anti-microbial, anti-tumor, anti-cancer, etc. It is known that the initiation of cancer usually takes several years and is related to unhealthy lifestyle, as well as dietary and environmental factors, such as stress, toxins and smoking. In fact, natural or synthetic substances have been used as cancer chemoprevention to delay, impede, or even stop cancer growing. This review is an attempt to collect current available phytochemicals from *Polyalthia species*, which exhibit anti-cancer potentials for chemoprevention purposes, providing directions for further research on the interesting agents and possible clinical applications.

## 1. Chemopreventive Concepts on Cancer Progression by Using Natural Products against Chronic Inflammation or Oxidative Stress

Cancer has become a chronic disease in modern societies, and the developments of precise personalized medicines and target therapies have been enlarged lately. Although some cancers may be curable, people still find some alternative strategies to prevent cancer progression. Chemoprevention was first introduced in 1976 and referred to the use of a natural or synthetic agent to reduce the risks and/or reverse cancer from developing [1]. The chemoprevention of cancer could be used in primary, secondary, and tertiary prevention pathways to use medicine or agents to prevent tumor formation in a healthy person, who has pre-cancerous lesions or already had cancer, respectively [2].

Collectively, studies have shown that chronic inflammation may be the initiation of cancer [3,4,5]. Thus, chemoprevention may include the concept of inhibition upon inflammation and oxidation to reverse the progress of carcinogenesis and ageing-induced gene mutation [5]. For example, aspirin (acetylsalicylic acid), a synthetic drug from the natural substance salicin, from myrtle and willow is a common prescription for its anti-pyretic, analgesic, and anti-platelet aggregation properties. It is accepted that aspirin at low doses triggers lipotoxins production to block cell proliferation and chronic inflammation, which may associate with a lower incidence and recurrence of polyps as well as reduce colon cancer risk [2].

Plants, microbes, animals, marines, and minerals are always the natural sources that scientists could discover new compounds for chemoprevention related to clinical therapeutics [6,7]. Recently, reports have demonstrated that dietary-derived flavonoids (genistein, rutin, epigallocatechin gallate, silmaylin, curcumin, resveratrol, etc.) exhibit distinct anti-oxidant, anti-inflammatory and anti-cancer activities [8]. Until now, only some substances have been approved by the Food and Drug Administration [7]. Nevertheless, people who advocate natural medicine or self-healing strategies against diseases may use plant extracts or herbal decoction as daily supplements to achieve the effectiveness of chemoprevention [6,8].

## 2. Polyalthia Genus Plants

**Figure 1:**
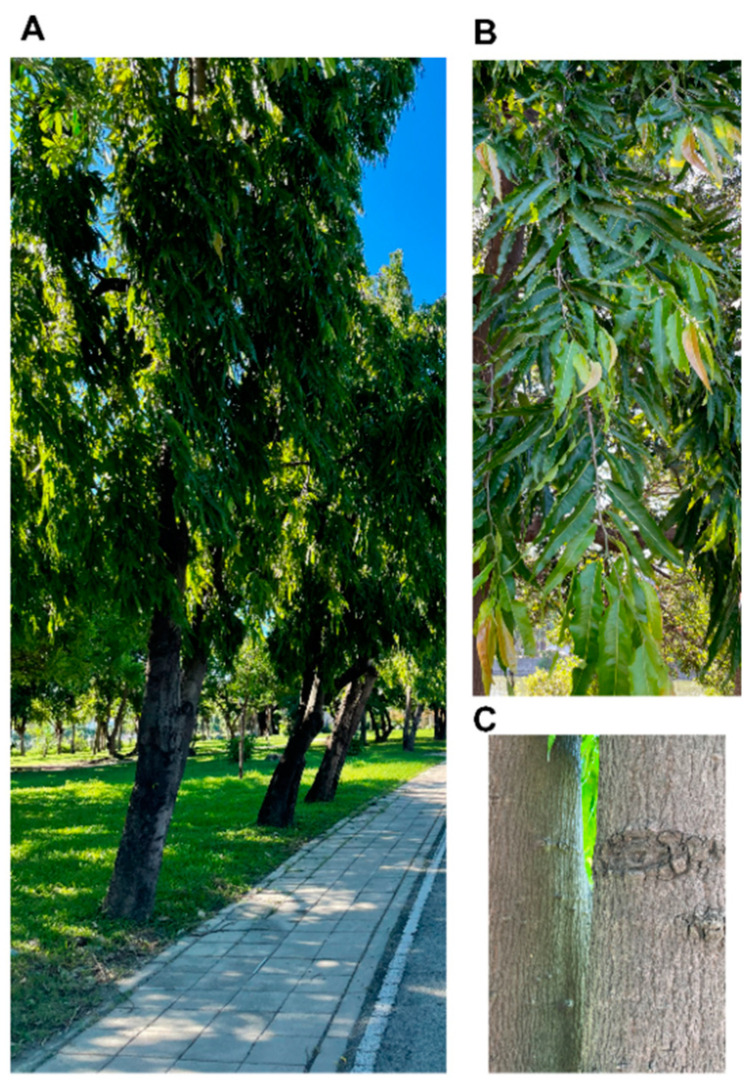
Photographs of *P. longifolia* Sonn. Thwaites pendula. (**A**) The whole tree, (**B**) leaves, and (**C**) stem bark of *P. longifolia*. Photos were shot at Kaohsiung, Taiwan, in 17 August, 2021.

The genus *Polyalthia* belongs to the Annonaceae family [9]. It is a type of flowering plant found in tropical and subtropical regions, including South Asia, South East Asia, and Australia [9]. According to the project of the world flora online webpage (http://www.worldfloraonline.org/ accessed on 28 July, 2021), the *Polyalthia* genus has 127 accepted species, consisting of trees, shrubs, and rare lianas [9]. In India, the *Polyalthia longifolia* is also called Ashoka or Indian mast tree due to its special appearance as a Stupa [10]. In Taiwan, it is commonly cultivated as landscape trees to avoid noise pollution (Figure 1A). *P. longfolia* is a tall (up to 12 m) and evergreen tree that grows symmetrically and produces green foliage (Figure 1B). The branches of the tree are peculiar, dropping down toward ground, giving the plant a narrow slender shape. These features make it readily available and is used in many folk medicines for the treatment of various ailments.

### Methods for Extraction of Phytochemical Compounds from Polyalthia

Through literatures review, the common method to extract the phytochemical compounds from species in *Polyalthia* is using organic solvent and followed by traditionally chromatographic techniques, such as column chromatography, high-performance liquid chromatography, etc. Because of the convenience and economic choice, most laboratories used methanol or ethanol as a polar protic solvent to prepare the crude extracts [11]. Methanol and water are better solvents to prepare plant decoction due to its high dielectric constants and dipole moments [11]. Additionally, the evaporation process is easier for methanol when compared to water. For example, the standardized extraction of *P. longifolia* was through adding dried samples (leaves (Figure 1B), twigs, flowers, fruits, barks (Figure 1C) and/or roots) to adequate volume of methanol and soaked the samples for 3-7 days at room temperature [11,12,13,14]. Filtrated samples were then concentrated by using a rotary evaporator and at 40–60 °C [12,13]. The concentrated extracts could be sterilized by filtration through a 0.22 µm membrane before further testing [11]. Finally, a thick, yellow-to-brownish-colour paste mass was the crude extracts of *P. longifolia*. The acute oral toxicity of the standardized extracts of *P. longifolia* leaves has been evaluated to be safe, and the dose can be used at 3240 mg/kg in Wistar albino rats [15] and at 5000 mg/kg in female Sprague-Dawley rats [16].

Generally, the discovery of the newly phytochemical compounds from natural sources is based on the bioactivity-guided fractionation, purification, and structure identification [17]. The fractions were then tested for their activities on the cytotoxic, anti-oxidant or anti-inflammatory effects. The active fractions will be chosen for further isolation of the bioactive compounds. Sometimes, the resolution of enantiomers is not easy and needs particular chromatographic columns to separate the distinct substances from each other [11,17]. In addition to the consumption of a large quantity of organic solvents, which may also raise the concerns of environmental pollution, the isolation and identification of the biochemical compounds from these fractions are time-consuming and labour-intensive processes that increase the difficulties of finding new compounds.

## 3. Phytochemical Constituents in Species of *Polyalthia*

Scientific reports on leaves, bark, stem bark, root, twigs, and seeds of *Polyalthia* have revealed dozens of types of alkaloids and terpenes with numerous biological and pharmacological activities with chemopreventive potentials, such as anti-bacterial [18,19,20], anti-fungal [21], anti-viral [22,23], anti-plasmodial [24,25,26], anti-inflammatory [27,28,29], anti-ulcer [30], anti-tumor [31,32,33], and anti-cancer [34,35,36] effects.

Literature reviews on recent works reveal that most abundant phytochemicals in *Polyalthia* plants are alkaloids and terpenes [37]. Other major bioactive phytochemicals in *Polyalthia* species are flavonoids, lignans, sterols, organic acids, etc. [27,38,39,40,41,42]. In fact, clerodane-type diterpenes may be one of the most well-studied and enriched-compound in *Polyalthia species*, and the pharmacological and physiological functions of 16-hydroxycleroda-3,13-dien-15,16-olide (36 and/or 38, abbreviated CD or HCD in literatures) have been studied by several groups [24,43,44,45,46,47].

CD, a major component of *P. longifolia* [14], has been validated to exhibit anti-microbial [24,48,49,50], anti-diabetic [51], anti-tumor [34,44,45,52], and anti-cancer [36,43] activities. Moreover, molecular docking studies have shown that 36 can be a multi-targets inhibitor to 3-hydroxy-3-methylglutaryl co-enzyme A (HMG-CoA) reductase [46], dipeptidyl peptidase 4 [51], focal adhesion kinase (FAK) [53], and phosphoinositide 3-kinase (PI3K) [45]. Besides, to compile the promising compounds that display chemopreventive activities, the molecular mechanisms of CD, one of the most potent agents isolated from *P. longifolia*, will be illustrated later.

## 4. Anti-Oxidant Phytochemicals in *Polyalthia*

**Figure 2:**
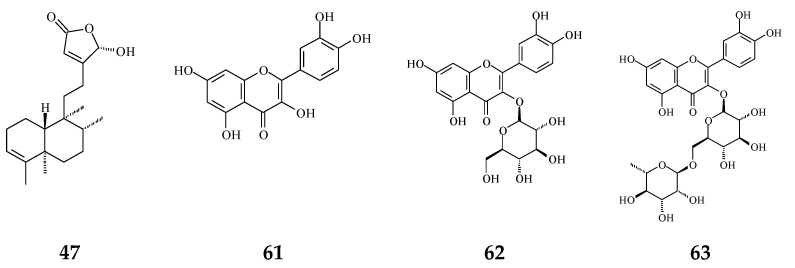
The phytochemical compounds isolated from species of *Polyalthia* with anti-oxidant activity (**47** and **61**–**63**).

Plant extracts and natural products are sources of anti-oxidative agents. As reported (Figure 2), flavonoids (**61**–**63**) [54,55] and proanthocyanidins [56] extracted from *P. longifolia* leaves and clerodane diterpenes (**47**) isolated from stem bark of *P. simiarum* [57] as well as stem bark of *P. longifolia* extracts [58] displayed anti-oxidative activities, as detected by the DPPH method and enzymatic activity assay.

Oyeyemi et al. (2020) demonstrated that *P. longifolia* aqueous and methanolic leaf extracts present the prophylactic and the curative activities against cadmium (a major environmental pollutant)-induced hepatotoxicity by relieving the oxidative stress in rats [59]. Moreover, phenol- and flavonoids-rich *P. longifolia* extracts have been demonstrated to improve paracetamol-treated rat liver injury related to free radicals [55]. In fact, Rai et al. (2019) have found that flavonoids from *P. longifolia* could block fructose-induced protein oxidation and glycation as well as the formation of advanced glycation end products [41]. Shih et al. (2010) have demonstrated that CD could ameliorate LPS-induced toxicity through the inhibition of redox signalling upon inducible nitric oxide synthase and gp91 (phox) in microglia cells [60]. All above studies suggest that the observed hepatoprotective and improvement of cell survival by *P. longifolia* extracts or clerodane diterpenes were related to their anti-oxidant activity.

Oxidation and anti-oxidation could be a double-edged sword, and the imbalance of redox signalling could cause oxidative stress [61]. It is well known that ageing, inflammation, environmental pollutants and ultraviolet radiation could promote to produce a large quantity of free radicals [61]. On the other hand, reactive oxygen species (ROS) overproduction has been contributed to an intrinsic apoptotic pathway in cancer research [62], which would trigger the release of cytochrome c from mitochondria, and then to induce caspase-9 and -3 cleavages, initiating cell apoptosis [62]. In fact, CD could promote ROS overproduction, which can be seen in some in vitro tumor cell lines [34,44,52]. One study has shown that CD enhanced ROS production, which concomitantly inhibited the activity of antioxidant enzymes, including superoxide dismutase, glutathione, glutathione peroxidase, and glutathione transferase in glioma cells [34].

## 5. Anti-Inflammatory Phytochemicals in *Polyalthia*

**Figure 3:**
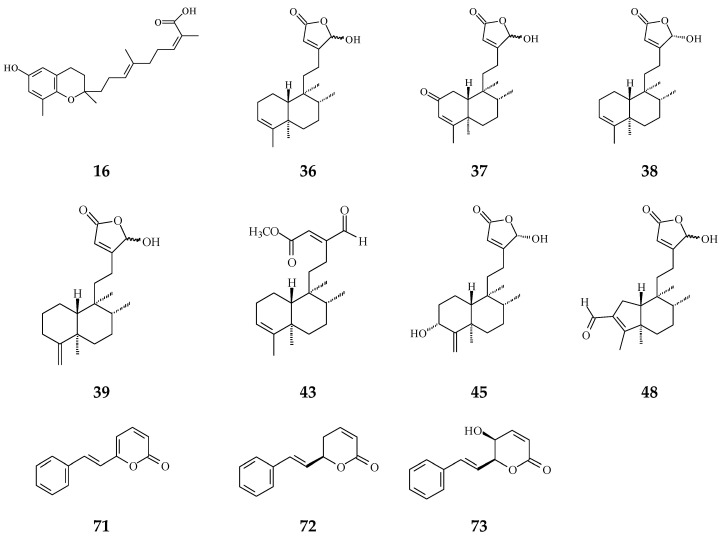
The phytochemical compounds isolated from species of *Polyalthia* with anti-inflammatory activity (**16**, **36**–**39**, **43**, **45**, **48** and **71**–**73**).

Inflammation plays a crucial role in carcinogenesis [3]. During tissue injury, a large number of cytokines and chemokines are attracted to the afflicted region to initiate and activate tissue-repairing processes [63]. Anti-inflammatory cytokines and pro-inflammatory cytokine signals are in balance, regulating normal inflammation conditions [63]. However, a growing body of evidence has shown that chronic inflammation or persistent infection are the main factors to induce tumor development [4,5]. Regardless of early in neoplasia formation or later in tumorigenic progress, inflammatory immune cells and the tumor itself would release many cytokines/chemokines and angiogenic factors, which would make a suitable microenvironment contributing to cancer deterioration [3]. The crude extracts of *Polyalthia* plants have been evaluated upon the anti-inflammation effects using in vitro and/or in vivo models. In Figure 3, several compounds from *Polyalthia* plants, including polycerasoidol (**16**) from *P. cerasoides* [28], **36**–**38**, **43**, **45**, and **48** from *P. longifolia* [27,64], and 6S-styryllactones (**71**–**73**) from *P. parviflora* leaves [65], have revealed the anti-inflammation activities.

Study has also shown that polycerasoidol (**16**) could decrease tumor necrosis factor alpha (TNFα-induced mononuclear cell adhesion to human umbilical endothelial cells at a concentration of 4.9 μM [28]. In addition, this prenylated benzopyran compound (**16**) was reported to be a dual peroxisome proliferator-activated receptor (PPAR)-agonists using in vitro activity assay plus prediction by molecular docking simulation, preventing cardiovascular events associated with metabolic disorders [28]. In addition to their anti-inflammatory function [66], PPAR and PPAR agonists have been shown to treat dyslipidaemia or type II diabetes, respectively [67], which could correlate to the phytochemicals in the *Polyalthia* genus with an anti-inflammatory characteristic. Indeed, dual PPAR agonists may combine both advantages to achieve more potent therapeutic application, which is ongoing in preclinical and clinical trials [68,69].

Anti-inflammatory, analgesic, and anti-pyretic drugs commonly used today are nonsteroidal anti-inflammatory drugs, which inhibit COX-2 activity and stop the downstream prostaglandin E2 (PGE2) production and the following inflammatory process [70]. The leaves, stem bark and root extracts of *P. longifolia* (300 mg/kgw) express higher activities against LPS-induced pyrexia than aspirin [71]. In fact, studies have demonstrated different effects of phytochemicals in the *Polyalthia* genus, such as that anti-inflammatory effects of 36 and 41 [64] have been authenticated on lipopolysaccharide (LPS)-treated RAW264.7 macrophages; 16-oxocleroda-3,13(14)*E*-dien-15-oic acid methyl ester (43) could inhibit formyl-l-methionyl-l-leucyl-l-phenylalanine/cytochalasin B (fLMP/CB)-induced superoxide anion generation in human neutrophils [27]; 36 could ameliorate LPS-induced nitric oxide (NO) production in RAW264.7 macrophages [64] as well as inhibit LPS-induced neurotoxicity through the down-regulation of COX-2 and NF-κB (p65) [60]; and the production of NO and inflammatory cytokines (PGE2, and TNFα) were all reversed by the 36 treatment [60].

The anti-inflammation activity of 16-hydroxycleroda-3,13*Z*-dien-15,16-olide (38), 16-hydroxycleroda-4(18),13-dien-15,16-olide (39), and 3,16-dihydroxycleroda-4(18),13(14)*Z*-dien-15,16-olide (45) have been determined by kinases inhibition assays upon cyclooxygenase-1 (COX-1) and -2 (COX-2) as well as 5-lipoxygenase (5-LOX) [29]. Specifically, 38 displays an excellent inhibition rate against COX-1 enzyme compared to indomethacin (COX-1 reference drug) at 10 μg/mL. It also shows better inhibition on 5-LOX enzyme than diclofenac (23.28 ± 0.31 nM). Furthermore, molecular docking and calculation binding affinities show that these two compounds are potent COX-1/2 and 5-LOX inhibitors [29], implying that both compounds could be possibly used for clinical application against inflammation as precise personalized medicines.

Furthermore, using an in vivo model, CD could improve azoxymethane/dextran sodium sulfate-induced IBD, which included a reduction in lymphocyte infiltration, lymphatic nodule enlargement, and shorter villi of the intestine [72]. Taken together, CD (36 and/or 38) could alleviate inflammation, which may link to COX-2 and NF-κB signaling pathways related to the suppression of pro-inflammatory cytokines and NO release.

## 6. Cytotoxic/Anti-Tumor Phytochemicals in *Polyalthia* and the Molecular Mechanism of CD-Induced Tumor Cell Death

**Figure 4:**
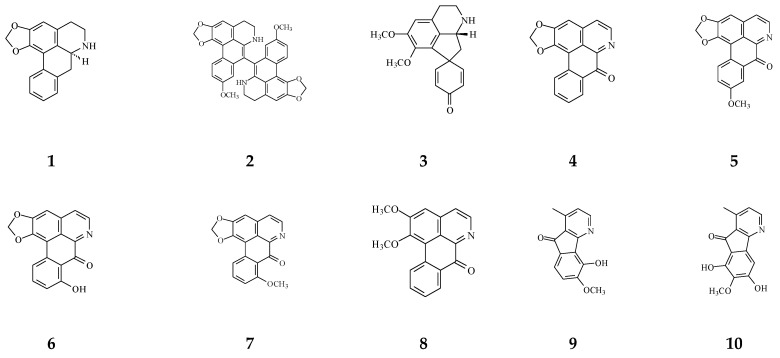

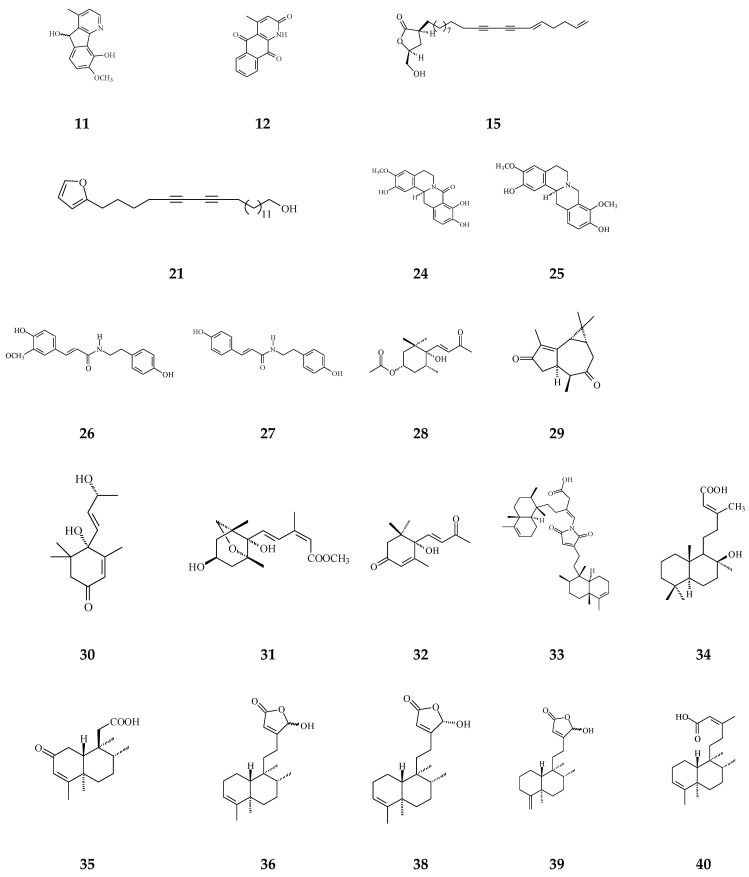

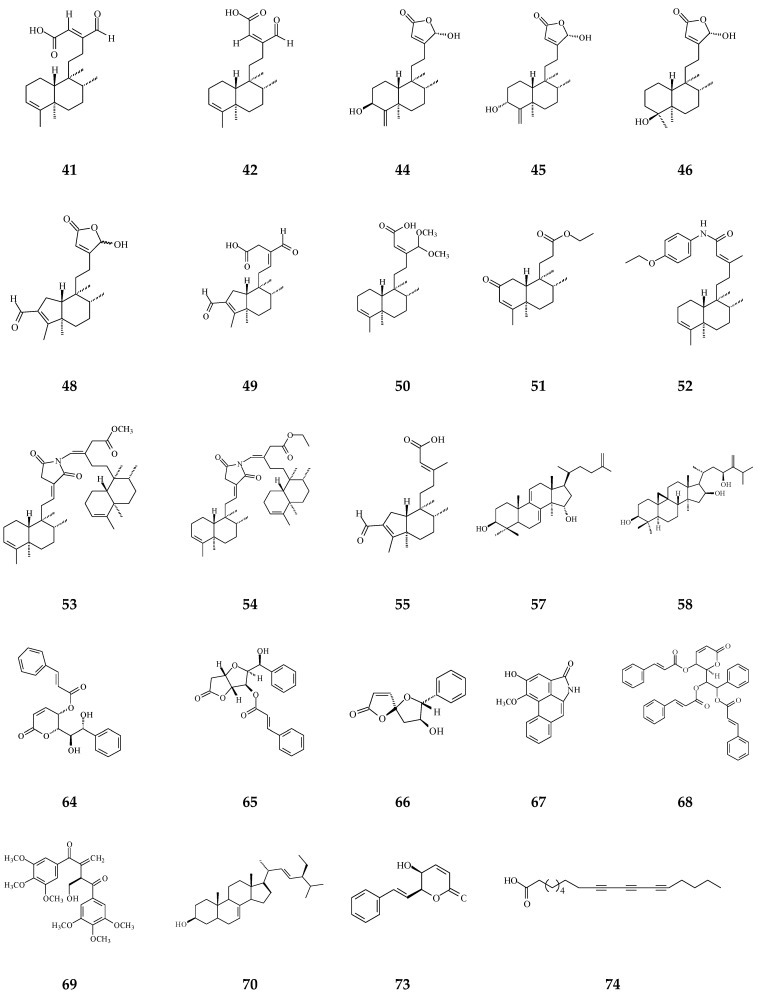
The phytochemical compounds isolated from species of *Polyalthia* with cytotoxic/anti-tumor activity (1–12, 15, 21, 24–36, 38–42, 44–46, 48–55, 57, 58, 64–70, 73, 74).

Cytotoxic compounds isolated from *Polyalthia* mainly belong to alkaloids and terpenes, which are summarized in Table 1 and Figure 4. By using survival tests (MTT or CCK-8), there are about 54 compounds exhibiting cytotoxic/anti-tumor effects, which show that IC_50_ values are in the range of nano-molar to micro-molar.

Eighteen alkaloid compounds, namely, (−)-anonaine (1), bidebiline E (2), (+)-stepharine (3), liriodenine (4), lanuginosine (oxoxylopine) (5), oxostephanosine (6), oxostephanine (7), Lysicamine (8), 5-hydroxy-6-methoxyonychine (isoursuline) (9), 6,8-dihydroxy-7-methoxy-1-methyl-azafluorenone (10), polylongine (11), marcanine A (12), debilisone E (15), 21-(2-furyl)heneicosa-14,16-diyne-1-ol(21), (−)-8-oxo-2,9,10-trihydroxy-3-methoxyberberine (consanguine B) (24), (−)-stepholidine (25), *N*-trans-feruloyltyramine (26), and *N*-trans-p-coumaroyltyramine (27), could induce cancerous cell death at a concentration of µg/mL or µM range. Compound 10 induces cell apoptosis in HL-60 through cleavage of caspase-8 and -9, indicating the activations of extrinsic and intrinsic caspase pathways. Besides, 10 has been tested on the adriamycin-resistant lung cancer cell line with an IC_50_ value of 3.6 µg/mL.

Twenty-seven terpene compounds, namely, polyalone A (28), 9-ketocyclocolorenone (29), blumenol A (30), (−)-methyl dihydrophaseate (31), bis-enone (32), longimide A (33), labd-13*E*-en-8-ol-15-oic acid (34), 1-naphthaleneacetic-7-oxo-1,2,3,4,4a,7,8,8a-octahydro1,2,4a,5-tetramethyl acid (35), 36, 16α-hydroxycleroda-3,13*Z*-dien-15,16-olide (38), 16-hydroxycleroda-4(18),13 -dien-15,16-olide (39), kolavenic acid (40), 16-oxocleroda-3,13*E*-dien-15-oic acid (41), 16-oxocleroda-3,13*Z*-dien-15-oic acid (polyalthialdoic acid) (42), 3β,16α-dihydroxycleroda-4(18),13(14)*Z*-dien-15,16-olide (44), (-)-3α,16α-dihydroxycleroda-4(18),13(14)*Z*-dien-15,16-olide (45), 4β,16α-dihydroxycleroda-13(14)*Z*-en-15,16-olide (46), (4→2)-abeo-16(R&S)-2,13*Z*-clerodadien-15,16-olide-3-al (48), (4→2)-abeo-2,13-diformyl-cleroda-2,12*E*-dien-14-oic acid (49), 16,16-dimethoxy-cleroda-3,13*Z*-dien-15-oic acid (50), polylauiester A (51), polylauiamide B (52), polylauiamide C (53), polylauiamide D (54), solidagonal acid (55), suberosol (57), and 24-methylenecycloartane-3β,16β,23β-triol (longitriol) (58), could reduce cell viabilities. Compounds 33, 37, 39, 41, 42, 44, 46, and 48 showed the best potency (IC_50_ values are below 5 µg/mL) against some tumor cell lines (Table 1).

Other natural products include crassalactone A (64), crassalactone B (65), crassalactone D (66), aristolactam AII (67), (+)-tricinnamate (68), (+)-rumphiin (69), -spinasterol (70), (−)-5-hydroxygoniothalamin (73), and octadeca-9,11,13-triynoic acid (74), which are not yet classified in the alkaloid or terpene family displaying cytotoxic effects against several tumor cell lines. Compounds 64–68 from *P. crassa* affect tumor cell growth at very low IC_50_ values in the range of 0.18–3.8 µg/mL [96]. However, the concentrations of these compounds may also hurt normal cells. Compounds 44 and 45 display cytotoxicity against both human tumor cell lines and the normal green monkey kidney epithelial cell line [77]. Longimide A (33) at a lower concentration (4.12–10.13 µg/mL) kills several tumor cell lines, while the cytotoxic concentration is 4 to 10-fold higher on the NIH-3T3 normal fibroblast cell line [83]. The IC_50_ value of longitriol (58) could inhibit breast and brain tumor cell proliferation and then induce apoptosis in colon cancer cell lines. At a concentration of 40.3 µM, 58 is also toxic on MRC-5 normal human fibroblasts. Therefore, the most toxic compound may not be a good agent for therapeutic application, which should be further authenticated by using an in vivo model.

Rupachandra and Sarada (2014) determined a fraction F2 purified from trypsin-treated *P. longifolia* seeds and found that F2 fraction caused A549 and HL-60 cell death through apoptosis [98]. They showed the average mass of this F2 fraction to be 679.8 *m/z* ratios by LC-ESI-MS/MS analysis; however, the exact structure was not shown [98].

There are many selective candidates for biological and pharmacological studies related to the accessibility of these specific compounds, which may need to be considered. In comparison to other cytotoxic alkaloids (milligram level output), the extraction of 9 kg of *P. longifolia* leaves could obtain about 12.2 g of 16-hydroxycleroda, 3,13-dien, 15,16 olide (38) [14] with a good yield rate, which does elevate its potentiality as a drug/medicine.

**Figure 5:**
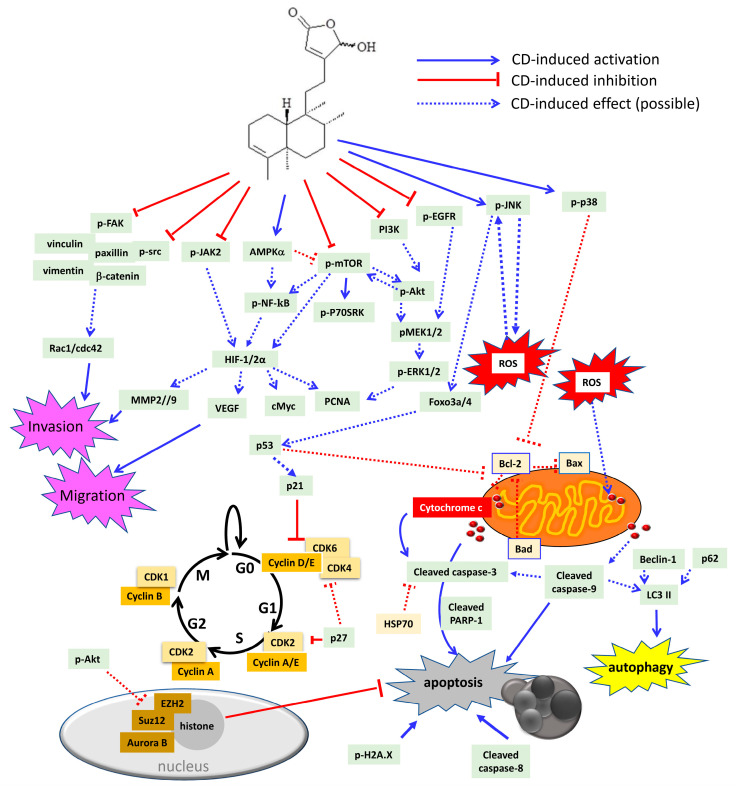
Schematic diagram of CD-induced possible molecular mechanisms in different tumor cells. CD could block cell proliferation through inactivation of several oncogenic molecules, including dephosphorylation of epidermal growth factor receptor (EGFR), PI3K, Akt, mammalian target of rapamycin (mTOR), ribosomal protein S6 kinase beta 1 (P70S6K), mitogen-activated protein kinase kinase 1/2 (MEK1/2), extracellular signal regulated kinase 1/2 (ERK1/2), and src. Besides, CD could also arrest cell cycle either at G0/G1 or G2/M phase through inhibition of cyclins and cyclin-dependent kinases (CDKs), as well as induction of the CDK inhibitor, p21, p27, and p53, respectively. In addition, CD could increase sub-G1 population, which indicates DNA fragmentation related to cell apoptosis. CD can trigger cell death via autophagy and/or apoptosis. CD has been shown to be involved with H2A.X phosphorylation as well as cleavage of caspase-3, -8, -9, and poly (ADP-ribose) polymerase-1 (PARP-1) to induce intrinsic and extrinsic apoptosis. Moreover, CD could promote ROS overproduction, which may induce cytochrome c release from the mitochondria outer membrane. The anti-apoptotic proteins that CD could suppress are heat shock protein 70 (HSP70) and B cell lymphoma 2 (Bcl-2). The pro-apoptotic proteins that CD could stimulate are Bad and Bax. Accordingly, CD induces phosphorylation of C-Jun N-terminal kinase (JNK), p38 MAPK, and 5’ AMP-activated protein kinase (AMPK). JNK has been linked to ROS generation, which may cause a positive feedback loop to further activate JNK itself. Activation of p38 MAPK has been demonstrated to reduce Bcl-2 expression and trigger the intrinsic apoptotic pathway. As a tumor suppressor, forkhead box O3 (foxo3a), foxo4 as well as p53 can be up-regulated by CD treatment. The induction of p53 by CD may cause CDK inhibitor p21 to impede cell cycle, or, on the other hand, induce Fas/caspase-8 and initiate extrinsic apoptotic cascade. CD could repress the polycomb repressive comb complex (PRC)2 by modulating enhancer of zeste homolog 2 (EZH2) and suppressor of zeste 12 homolog (Suz12) as well as regulating histone demethylation to induce apoptosis. The inflammatory signalling pathway includes Janus kinase 2 (JAK2) and NF-κB, which could be both inactivated by CD treatment. CD could abrogate hypoxia inducible factor (HIF) 1 and 2 expression. Additionally, the HIF-downstream molecules, such as cMyc, vascular endothelial growth factor (VEGF), and matrix metalloproteinases (MMP2 and MMP9), could all be down-regulated by CD. Moreover, CD can induce cancer cell anoikis by dephosphorylation of the FAK pathway to abolish FAK-associated proteins, including vinculin, paxillin, vimentin, β-catenin, and src kinase. Rac1 and cdc42 protein expression are also down-regulated by CD.

At the concentration of 20–40 µM, CD (36) reduced tumor cell proliferation in solid tumors, such as glioma [34,43], glioblastoma [45], urothelial [44,52,100], breast [27], colon [45,72], lung [45], hepatoma [72], and head and neck [36] carcinoma cell lines, and in liquid tumors (leukemia) [45,86,99], respectively. Among these isolated compounds from *Polyalthia*, CD is the well-studied compound in anti-cancer fields. Accordingly, CD could become a potential agent, which may target multiple signalling molecules, including oncogenic, inflammatory, migratory, and invasive pathways (Figure 5 and Table 2).

Oncogenic pathways, such as mitogen-activated protein kinases (MAPKs) and PI3K/Akt signalling cascades, mainly contribute to tumor proliferation and cell survival [101]. Inhibitions of ERK1/2 and/or PI3K/Akt pathways by CD would suppress tumor cell proliferation and tumor growth, which have been well investigated and illustrated in RCC [44] and bladder cancer cell lines [52]. However, up-regulation of C-Jun N-terminal kinase (JNK), p38 MAPK, and ERK1/2 phosphorylation, concomitantly with the induction of apoptosis by CD, was also seen in glioma and leukemia cell lines [34,45]. In fact, CD could inhibit cell proliferation through cell cycle arrest at the G2/M phase [44,45] or G0/G1 phase [34,36,52]. Moreover, tumor suppressor proteins, such as p53, FoxO3a and FoxO4, could be increased by CD in RCC [44] and leukemia cancer cell lines [45] to induce cell apoptosis. Stress-activated JNKs, p38 MAPK, and ERK are double-face kinases in regulating cell death and survival, and this may because of a complex cross-talk network and/or a positive feedback loop exhibiting in cells [102]. Reports have shown that ROS generation activates JNK/p38 MAPK, which in turn induces ROS elevation in a feedback loop [103,104,105].

It is well accepted that NF-κB serves as an essential moderator in modulating inflammation through induction of pro-inflammatory cytokines [106]. Study has shown that transcription factor NF-κB would have cross-talk with other signalling pathways, such as FAK, mTOR or PI3K/Akt [106], to regulate inflammation, and the inhibition of pNF-κB by CD did affect inflammation response in colon cancer [72] cell line. Accordingly, it is highly possible that CD could alleviate inflammatory bowel disease (IBD), which may also relate to the inactivation of the NF-κB pathway.

Beside the inactivation of those proliferative and pro-inflammatory signaling cascades, Shanmugapriya et al. (2019 and 2020) reported that polyphenol-rich *P. longifolia* extracts induced HeLa cell apoptosis by down-regulation of oncogenic micro-ribonucleic acid (miRNA)-221-5p in HeLa cells [107,108]. MicroRNA is a non-coding small RNA, which consists of about 21-25 nucleotides in length and is base-pairing to the targeted messenger RNA (mRNA) [109]. The major function of miRNA appears to repress gene expression by the inhibition of translation, promotion of mRNA cleavage, and deadenylation [109]. A single miRNA is able to control up to hundreds (or more) mRNA; therefore, any mis-expression or mis-regulation of miRNA could lead to the development of tumor cells [110]. In summary, more studies are required to further clarify the relationship between the miRNAs and the silenced genes under the administration of crude extracts or an isolated single compound in *Polyalthia*.

## 7. Anti-Cancer Potential of *Polyalthia* Genus

Although there are hundreds of chemicals isolated from species of *Polyalthia*, few studies illustrate the investigation on anti-cancer activity of the single compound or *Polyalthia* extracts. Afolabi et al. (2020) provide the evidence that methanol extracts of *P. longifolia* exhibited anti-cancer activity against metastatic prostate cancer [35]. This study shows that methanol extracts of *P. longifolia* promoted the activation of endoplasmic reticulum stress and induced intrinsic apoptotic pathways [35]. Through proteomic and biochemical analysis, the glucose-regulated protein 78 (GRP78/BiP) was determined as a crucial starter to initiate ER stress and induce cell apoptosis [35]. One of the possible compounds that lead to impede prostate cancerous cell growth may be the tetranorditerpene 1-naphthalene acetic-7-oxo-1,2,3,4,4a,7,8,8a-octahydro-1,2,4a,5-tetramethyl acid (35) [35], which could also inhibit human leukemia HL-60 cell proliferation [84].

CD (38) has been determined as a new structural class of HMG-CoA reductase inhibitor [46] that alleviates adipogenesis in vitro and in vivo [47]. An FDA-approved drug, statin, a well-known inhibitor of HMG-CoA reductase, is now undergoing clinical trials for combination with standard chemotherapy or with other molecular-targeted drugs to improve cancer patients’ treatment outcomes and overcome drug resistance [111]. Velmurugan et al. (2018) reported that CD (36) enhances tamoxifen-induced apoptosis in both MCF-7 and MDA-MB-231 human breast cancer cells [112]. It is expected to soon have the investigation of HMG-CoA reductase-mediated molecular mechanism by CD treatment on some aberrant sterol metabolic cancer subtypes, such as ER-negative breast cancer and RCC.

Lin et al. (2011) demonstrated that CD is an inhibitor for PI3K. Moreover, the CD-inactivated Akt pathway may link to suppress the PRC2 complex and to reactivate downstream tumor suppressor gene expression. They also demonstrated that CD potentiates imatinib-induced cell death in K562, T315I-Ba/F3, SW620, and A549 cell lines. Taken together, CD may possibly be developed in combination with other clinical agents for tumor treatments.

Cheng et al. (2016) demonstrated that CD inhibited head and neck cancer growth by using the xenograft model, which showed that the effective intraperitoneal injection dosages were 6.5 and 19.5 mg/kgw/2 day by a seven-round treatment course [36], respectively. CD, like many other natural products, is insoluble in water. The poor bioavailability limits its effectiveness and usefulness in clinical therapeutics. The same group showed that enteric-coated nanoparticles of CD with intraperitoneal injection displayed more potently effective dosage with 0.16 mg/kgw/daily for a 10-day treatment period [43].

Hussain et al. (2018) evaluated a semi-synthetic diterpenoid, 16(R&S) phenylamino-cleroda-3,13(14)*Z*-dien-15,16 olide (derived from 16-oxocleroda-3,13(14)*E*-dien-15-oic acid (41), which could inhibit neuroblastoma SH-5Y5 cell proliferation through modulating the p53 pathway and apoptosis [113]. The IC_50_ of this semi-synthetic compound is 12.5 µM for 48 h of treatment in SH-5Y5 cells, which could be comparative with cisplatin administration [113]. Additionally, authors suggested that this agent did not affect the renal system in vivo, which could be considered for further cancer treatment.

## 8. Chemoprevention Potential of Phytochemical Compounds from *Polyalthia*

### 8.1. Phytochemical Compounds with Anti-Bacterial and Anti-Fungal Activities

**Figure 6:**
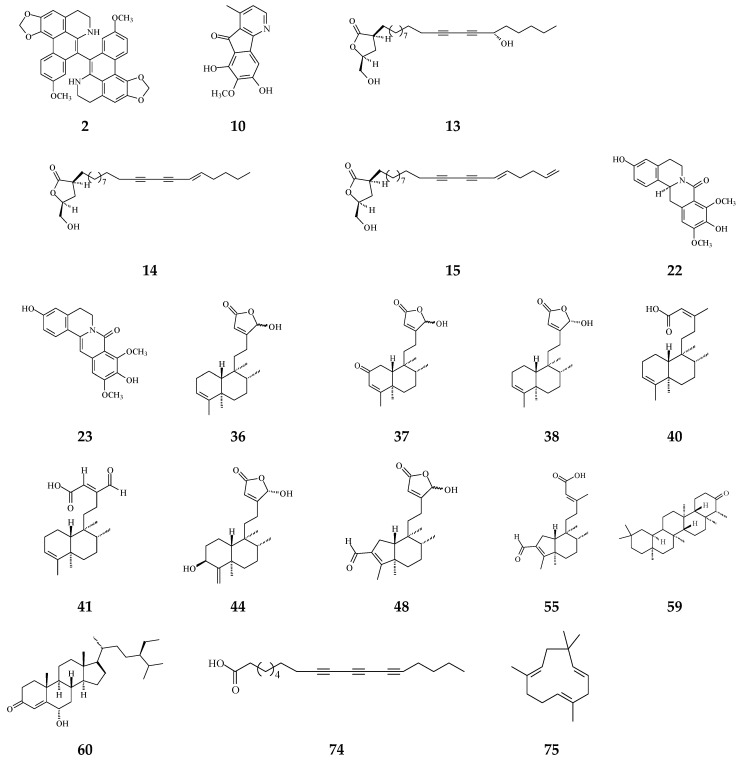
The phytochemical compounds isolated from species of *Polyalthia* have anti-bacterial (2, 10, 13–15, 22, 23, 36–38, 40, 41, 44, 48, 55, 59, 60, 74, and 75) and anti-fungal (36 and 41) activities.

It has been known that certain types of infection are linked to about 13% of all cancer cases [114]. Anti-bacterial and anti-fungal compounds from species of *Polyalthia* are listed in Table 1 and the structures are shown in Figure 6. Alkaloids and terpenes are origins of anti-microbial agents in *Polyalthia* (Table 1). Bidebiline E (2), 6, 8-dihydroxy-7-methoxy-1-methyl-azafluorenone (10), debilisone B (13), debilisone C (14), debilisone E (15) as well as natural products octadeca-9,11,13-triynoic acid (74) and -humulene have been shown to express potent inhibition against *M. tuberculosis* (Table 1). Among these compounds, 10 showed the highest potency (MIC 0.78 µg/mL) [75]. Pendulamine A (22) and pendulamine B (23) are classified in 8-oxoprotoberberine, showing broad spectrum inhibitory activity against Gram-positive and Gram-negative bacteria [18]. The MIC in the range of 0.02–20 µg/mL against the tested bacteria [18]. The authors suggested that the anti-bacterial activity is associated with compounds owing a monosubstituted A ring with a hydroxyl group at C-3 [18].

The phytochemical compounds of clerodane diterpenoids 36-38, 16-oxocleroda-3,13*E*-dien-15-oic acid (41), 3β,16α-dihydroxycleroda-4(18),13(14)*Z*-dien-15,16-olide (44), (4→2)-abeo-16(R&S)-2,13Z-clerodadien-15,16-olide-3-al (48), solidagonal acid (55), friedelin (59), and stigmast-4-ene-6α-ol-3-one (60) also display anti-bacterial activity against both Gram-positive and Gram-negative bacteria (Table 1). The tertiary chemoprevention of cancer is aimed to prevent cancer recurrence or second tumor/cancer formation in those who have already suffered from curative treatment [2]. Gram-positive and Gram-negative bacteria are known to affect or deteriorate cancer patients’ postoperative recovery [114]. Kolavenic acid (40) only kills Gram-positive bacteria [85]. Compound 38 shows the best potency with MIC at 0.78 µg/mL against Gram-negative bacteria (*E. coli*, *P. aeryginosa*, and *S. typhimurium*) [20]. On the other hand, 36 and 41 exhibited anti-fungal activity with moderate MIC in the range of 62.5–250 µg/mL [85]. 16-Hydroxycleroda-3,13Z-dien-15,16-olide (36), 38, 59, and 60 displayed better potency against Gram-positive bacteria with MIC in the range of 1.56–7.8 µg/mL (Table 1).

**Figure 7:**
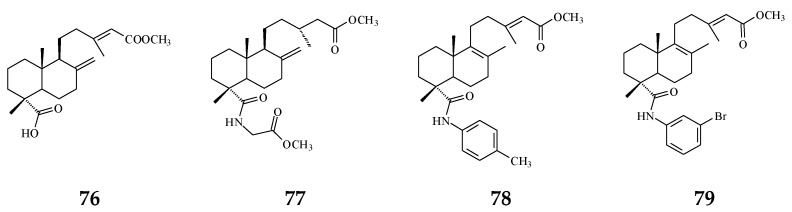
The synthetic compounds from (4*S*,9*R*,10*R*) methyl 18-carboxy-labda-8,13(*E*)-dien-15-oate (76) pose anti-ulcer activity (76–79).

The bacterium *Helicobacter pylori* infection is responsible for approximately 90% gastric cancer worldwide [115]. Edmond et al. (2020) reported that 36 and (4→2)-abeo-16(*R*&*S*)-2,13*Z*-clerodadien-15,16-olide-3-al (48) are potent agents against *H. pylori*, and the MIC are 31.25 and 125 µg/mL, respectively, compared with IC_50_ of the reference drug clarithromycin of 1.95 µg/mL [116]. *H. pylori*-induced gastric carcinogenesis is strongly associated with chronic inflammation [115]. Compound 36 (or 38) and 48 display better activity against histamine release at the concentration of 29.7 and 189.2 µg/mL, compared with diclofenac ‘s IC_50_ of 17.9 µg/mL [116]. The authors concluded that the weaker activity of 48 are due to (4→2)-abeo migration in it [116]. Study showed that Labdeneamides (77–79) from (4*S*,9*R*,10*R*) methyl 18-carboxy-labda-8,13(*E*)-diene-15-oate (76), isolated from *P. macropoda*, also expressed anti-ulcer activity against ethanol/HCl-induced gastric mucosa lesions [30]. The compounds 77–79 (Figure 7) showed the excellent anti-ulcer activity at a single oral dose of 0.1 mg/kgw [30]. *H. pylori* and gastric mucosa ulcer are the high-risk factors related to gastric cancer [3,115]. Thus, it is highly recommended that the crude extracts and/or CD may be used to possibly treat gastric cancers.

### 8.2. Phytochemical Compounds with Anti-Viral Activity

**Figure 8:**
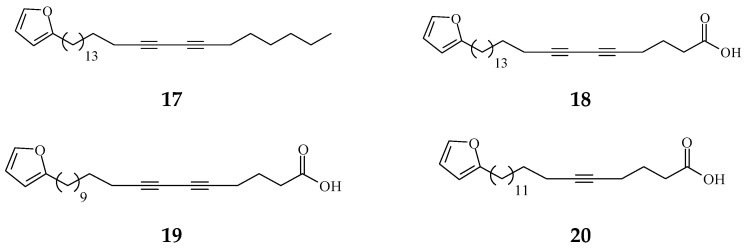

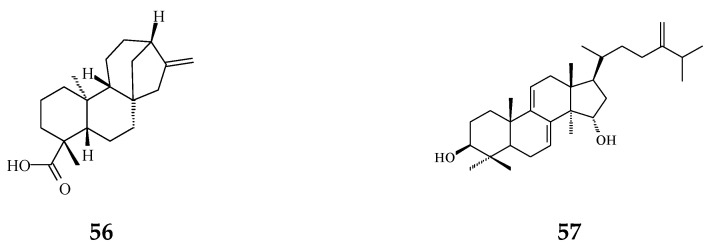
The phytochemical compounds isolated from species of *Polyalthia* exhibit anti-viral activity (17–20, 56, and 57).

Viruses cause cancer by infection and alteration of genetic codes of host immune cells when the immune system is suppressed or weakened [114]. Three alkaloids and two terpene phytochemical compounds exhibit anti-viral activity (Figure 8). Prenylated benzopyran 1-(2-furyl)pentacosa-16,18-diyne (17) and 23-(2-furyl)tricosa-5,7-diynoic acid (18), terpenes ENT-kaur-16-en-19-oic acid (56) and suberosol (57) inhibit HIV reverse transcriptase activity and viral syncytium. The 2-subsitute furan, 19-(2-furyl)nonadeca-5,7-diynoic acid (19) and 19-(2-furyl)nonadeca-5-ynoic acid (20) also exhibit anti-viral activity against HSV-1 virus.

## 9. Conclusions and Future Perspectives

The *Polyalthia* genus is a resourceful plant, which can be found across the whole island of Taiwan. In fact, numerous kinds of chemical compounds and secondary metabolites from *Polyalthia* have been studied, showing pharmacological activities that illustrate its values. However, more in vitro and in vivo mechanism investigations would be needed to better understand how it works with the pharmacological effects. Furthermore, the pure components inside *Polyalthia* with pharmacological effects should be additionally examined to possibly find more effective substances. Certainly, for the known pure compounds, such as 16-hydroxycleroda-3,13-dien-16,15-olide (36) and/or 16α-hydroxycleroda-3,13-dien-16,15-olide (38), it should be worth further investigating in detail in vitro and in vivo mechanisms that can conceivably be used as drugs for chemoprevention. 

## Figures and Tables

**Table 1:** Bioactive compounds isolated from *Polyalthia* plants.

Category	Name of Compound	Pharmacological Activities Described in References	Concentration	In Vitro/ In Vivo Model
			IC_50_/EC_50_/	
			Minimal Inhibitory Concentration (MIC)	
**Aporphine**	(−)-Anonaine (1)	Cytotoxicity [14]	8.6–28.9 μM	AGS, DLD1, HA59T, and HepG2
	Bidebiline E (2)	Anti-bacterial [19]	6.25 μg/mL	*Mycobacterium tuberculosis*
		Inhibition of wnt protein [73]	20.2 μM	SW480
**Proaporphine**	(+)-Stepharine (3)	Cytotoxicity [27]	9.4–9.9 μg/mL	MCF-7, MDA-MB-231
**Oxoaporphine**	Liriodenine (4)	Cytotoxicity [27,31,74]	4.46–10.28 μg/mL	MCF-7, MDA-MB-231
			0.57–2.33 μg/mL	KB, A549, HCT-8, P-388, and L-1210,
	Lanuginosine (oxoxylopine) (5)	Cytotoxicity [74]	1 μg/mL	Unavalible
	Oxostephanosine (6)	Cytotoxicity [74]	1 μg/mL	Unavalible
	Oxostephanine (7)	Cytotoxicity [39]	1.47–1.73 μg/mL [39]	SPC-A-1 and BEL-7402
	Lysicamine (8)	Cytotoxicity [27]	8.94–16.75 μg/mL	MCF-7, MDA-MB-231
**Azafluorene**	5-Hydroxy-6-methoxyonychine (isoursuline) (9)	Cytotoxicity [14]	21.7 μg/mL	HA59T
	6,8-Dihydroxy-7-methoxy-1-methyl-azafluorenone (10)	Cytotoxicity [75]	2.64–3.58 μg/mL	A549, GLC4, and adrinamycin-resistance GLC4
		Apoptosis [76]	20–55 μM	HL-60, U937, MOLT-4, MDA-MB-231, and HepG2
		anti-bacterial [75]	0.78 μg/mL	*M. tuberculosis*
	Polylongine (11)	Cytotoxicity [27]	9.94–10.41 μg/mL	MCF-7 and MDA-MB-231
**Anthraquinones**	Marcanine A (12)	Cytotoxicity [77]	1.53–11.78 μM	BEL-7402, K562, SPCA-1, and SGC-7409
**Acetogenin**	Debilisone B (13)	Anti-bacterial [78]	*25* μg/mL	*M. tuberculosis*
	Debilisone C (14)	Anti-bacterial [78]	12.5 μg/mL	Same as above
	Debilisone E (15)	Anti-bacterial [78,79]	25 μg/mL	*M. tuberculosis*
			64 μg/mL	*Morexella catarrhalis*
		Cytotoxicity [79]	18.4–40.3 μg/mL	HepG2, A549, HCC-S102, HL-60, and P-388
**Prenylated Benzopyran**	Polycerasoidol (16)	Anti-inflammatory [28]	4.9 μΜ	Inhibition of mononuclear leukocyte adhesion to endothelium
	1-(2-furyl)pentacosa-16,18-diyne (17)	Anti-viral [80]	43.3 μg/mL	ΔTat/RevMC99 syncytium assay for human immunodeficiency virus (HIV)
	23-(2-furyl)tricosa-5,7-diynoic acid (18)	Anti-viral [80]	8.9 μg/mL	Same as above
**2-substituted furans**	19-(2-furyl)nonadeca-5,7-diynoic acid (19)	Anti-viral [22]	47 μg/mL	Anti-viral assay against herpes simplex type 1 (HSV-1)
	19-(2-furyl)nonadeca-5-ynoic acid (20)	Anti-viral [22]	19.4 μg/mL	Same as above
	21-(2-furyl)heneicosa-14,16-diyne-1-ol (21)	Cytotoxicity [22]	12.4 μg/mL	NCI-H187
**8-Oxoprotoberberine**	pendulamine A (22)	Anti-bacterial [18]	0.02 μg/mL	Gram-positive bacteria
				*Corynebacterium hoffmanii* and *Micrococcus lysodicklycus*
			0.2 μg/mL	*Staphylococcus aureus*
			2 μg/mL	*Bacillus subtillis*
			12.5 μg/mL	*Streptococcus viridans*
			20 μg/mL	*S. pyogenes*
			0.02 μg/mL	Gram-negative bacteria
				*Salmonella typhi*
			0.2 μg/mL	*S. paratyphi A*
			2 μg/mL	*Klebsiella pneumoniae* and *Pseudomonas aeruginosa*
	pendulamine B (23)	Anti-bacterial [18]	0.02 μg/mL	Gram-positive bacteria
				*C. hoffmanii*, *S. viridans*, and *M. lysodicklycus*
			0.2 μg/mL	*S. aureus*,
			2 μg/mL	*S.s faecalis*
			20 μg/mL	*S. pyogenes*
			0.2 μg/mL	Gram-negative bacteria
				*S. paratyphi A and S. typhi*
			2 μg/mL	*K. pneumoniae*
	(−)-8-oxo-2,9,10-Trihydroxy-3-methoxyberberine	Cytotoxicity [81]	24.1 μM	MCF-7
	(consanguine B) (24)		33.5 μM	HeLa
**Tetrahydroprotoberberine**	(−)-stepholidine (25)	Cytotoxicity [27]	16.56 μg/mL	MCF-7
**Amides**	*N*-trans-Feruloyltyramine (26)	Cytotoxicity [27]	21.17-25.54 μg/mL	MCF-7, MDA-MB-231, HepG2, Hep3B
	*N*-trans-p-Coumaroyltyramine (27)	Cytotoxicity [27]	17.35 μg/mL	MCF-7
**Sesquiterpenes**	Polyalone A (28)	Cytotoxicity [82]	18.9–24.8 μΜ	HeLa, A549, MCF-7, and HL-60
	9-Ketocyclocolorenone (29)	Cytotoxicity [82]	20.5–26.2 μΜ	Same as above
	Blumenol A(30)	Cytotoxicity [82]	24.5–28.2 μΜ	Same as above
	(−)-Methyl dihydrophaseate (31)	Cytotoxicity [82]	22.6–27.1 μΜ	Same as above
	Bis-enone (32)	Cytotoxicity [82]	25.6–30.1 μΜ	Same as above
**Diterpenoids**	Longimide A (33)	Cytotoxicity [83]	4.12–10.13 μg/mL	KB, MCF-7, A549, and C33A
			44.7 μg/mL	NIH3T3
	labd-13*E*-en-8-ol-15-oic acid (34)	Cytotoxicity [27]	15.4–18.33 μg/mL	HepG2 and Hep3B
	1-naphthaleneacetic-7-oxo-1,2,3,4,4a,7,8,8a-octahydro1,2,4a,5-tetramethyl acid (35)	Cytotoxicity [84]	50 μM	HL-60
**Clerodane diterpenoids**	16-Hydroxycleroda-3,13*Z*-dien-15,16-olide (36)	Cytotoxicity, apoptosis, anti-cancer [36,43]	Details are in Table 2	786-O, A498, HL-60, T24, C6, N18, Caco-2, K562, MCF-7, MDA-MB-231, GBM8401,
				SW620, MOLT-4, HepG2, Hep3B, and A549
		Anti-inflammatory [27,60,72]	3.05 ± 1.13 μg/mL	Inhibition of fMLP/CB-induced superoxide anion production by human neutrophils.
			1–10 μM	LPS-treated RAW264.7 cells
			1.6–6.4 mg/kg	irritable bowel disease in mouse model (ADM/DSS-induced colitis)
		Anti-bacterial [85]		Gram-negative bacteria
			125 μg/mL	*S. typhi*
			250 μg/mL	*P. aeruginosa, K. ozaenae, and Proteus mirabilis*
				Gram-positive bacteria
			7.8 μg/mL	*S. aureus*
			125 μg/mL	*S. pyogenes*
			500 μg/mL	*C. hoffmanii*
		Anti-fungal [85]	62.5 μg/mL	*Aspergillus niger* and *Trichophyton metagrophyte*
			250 μg/mL	*Candida albicans*
	16-Hydroxy-cleroda-3,13(14)*Z*-dien-15,16-olide-2-one (37)	Anti-bacterial [85]		Gram-positive bacteria
			15.6 μg/mL	*B. subtilis*
		Anti-inflammatory [27]	62.5 μg/mL	*C. diphtheriae*, *C. xerosis*, and *S. aureus*
			500 μg/mL	*C. hoffmanii*, and *S. pyogenes*
			7.96 ± 1.78 μg/mL	Inhibition of fMLP/CB-induced superoxide anion production by human neutrophils
	16α-Hydroxycleroda-3,13*Z*-dien-15,16-olide (38)	Cytotoxicity [14,33,84,86,87,88],	0.5 μg/mL	P-388
			1.2 μg/mL	Mel2
			3.4–8.7 μg/mL	A431, BC1, Col2, HT, KB, drug-resistance KB, LNCaP, Lu1, and ZR75-1
			23.6–26.9 μM	AGS, HA59T
			21.28–34.89 μM	HT-29
				A549, MCF-7, HL-60, SMMC-7721, and SW-480
		Anti-bacterial [20,48,49,89]		Gram-positive bacteria
			6.25 μg/mL	*S. aureus*, *Sporothrix schenckii*, and *Arthrobacter citreus*
			1.56 μg/mL	*B. subtillis*, *B. polymyxa*, and *B. pumilus*
			3.12 μg/mL	*B. cereus, B. licheniformis, and Clostridium* sp.
				Gram-negative bacteria
			0.78 μg/mL	*E. coli*, *P. aeryginosa*, and *S. typhimurium*
			1.56 μg/mL	*K. aerogenes* and *Sarcina lutea*
			3.12 μg/mL	*P. putida* and *Nocardia* sp.
		Anti-inflammatory [29]	9.46 ± 0.33 nM	COX1 inhibitory assay
			10.34 ± 0.26 nM	COX2 inhibitory assay
			14.38 ± 0.32 nM	5-LOX inhibitory assay
	16-Hydroxycleroda-4(18),13 -dien-15,16-olide (39)	Cytotoxicity [27]	1.97–10.43 μg/mL	MCF-7, MDA-MB-231, HepG2, and Hep3B
		Anti-inflammatory [29]	11.85 ± 0.19 nM	COX1 inhibitory assay
			8.49 ± 0.55 nM	COX2 inhibitory assay
			14.38 ± 0.32 nM	5-LOX inhibitory assay
	Kolavenic acid (40)	cytotoxicity [87]	1.39–3.34 μg/mL	A549, MCF-7, and HT-29
		Anti-bacterial [85]	31.25 μg/mL	*B. subtilis* and *C. diphtheriae*
			125 μg/mL	*C. hoffmanii* and *C. xerosis*
	16-Oxocleroda-3,13*E*-dien-15-oic acid (41)	Cytotoxicity [10]	3.1–3.7 μM	MCF-7 and A549
		anti-bacterial [85]		Gram-negative bacteria
			500 μg/mL	*P. aeruginosa, S. typhi, K. ozaenae, K. aerogenes, E. coli, sarcina lutea, Nocardia sp., and P. mirabilis*
				Gram-positive bacteria
			500 μg/mL	*A. citreus, B. cereus, B. licheniformis, B. polymyxa, B. pumilus, B. subtilis, Clostridium* sp. *S. pyogenes*, *C. hoffmanii*, and *S. aureus*
		Anti-fungal [85]	62.5 μg/mL	*Trichonphyton mentagrophyte*
			125 μg/mL	*A. niger*
			250 μg/mL	*C. albicans*
	16-Oxocleroda-3,13*Z*-dien-15-oic acid (polyalthialdoic acid) (42)	Cytotoxicity [87]	0.552–0.753 μg/mL	A549, MCF-7, and HT-29
	16-Oxocelroda-3,13(14)*E*-dien-15-oic acid methyl ester (43)	Anti-inflammatory [27]	0.6 ± 0.09 μg/mL	Inhibition of fMLP/CB-induced superoxide anion production by human neutrophils
	3β,16α-dihydroxycleroda-4(18),13(14)*Z*-dien-15,16-olide (44)	Cytotoxicity [33]	2.2–16μg/mL	P-388, BC1, Col2, LNCaP, Lu1, ZR75-1
			12–16 μg/mL	Mel2, A431, HT, and KB
		Cytotoxicity [90]	10.474–24.096 μg/mL	KB, C33A, PA1, MCF-7
			18.564 μg/mL	Vero
		anti-bacterial [85]		Gram-positive bacteria
			62.5 μg/mL	*C. diphtheriae*, *C. xerosis*, and *S. pyogenes*
			125 μg/mL	*S. faecalis* and *C. hoffmanii*
			250 μg/mL	*S. saprophyticus*
			500 μg/mL	*B. subtilis*
	(−)-3α,16α-dihydroxycleroda-4(18),13(14)*Z*-dien-15,16-olide (45)	Cytotoxicity [90]	13.415–29.778 μg/mL	KB, C33A, PA1, and MCF-7
			20.345 μg/mL	Vero
		Anti-inflammatory [29]	10.85 ± 0.17 nM	COX1 inhibitory assay
			12.82 ± 0.21 nM	COX2 inhibitory assay
			16.94 ± 0.56 nM	5-LOX inhibitory assay
	4β,16α-dihydroxycleroda-13(14)*Z*-en-15,16-olide (46)	Cytotoxicity [33]	5.1–16 μM	A431. BC1, Col2, HT, LNCaP, Lu1, Mel2, P-388, ZR75-1
	16β-Hydroxycleroda-3,13(14)*Z*-dien-15,16-olide (47)	Anti-oxidant [57]	23.5 μg/mL	DPPH assay
	(4→2)-abeo-16(R&S)-2,13*Z*-clerodadien-15,16-olide-3-al (48)	Cytotoxicity [27]	2.36–11.89 μg/mL	MCF-7, MDA-MB-231, HepG2, and Hep3B
		Anti-bacterial [85]		Gram-positive bacteria
			31.25 μg/mL	*B. subtilis*
			125 μg/mL	*C. hoffmanii, C. xerosis, S. saprophyticus, S. faecalis, and S. pyogenes*
		Anti-inflammatory [27]	4.32 ± 0.59 μg/mL	Inhibition of fMLP/CB-induced superoxide anion production by human neutrophils
	(4→2)-abeo-2,13-diformyl-cleroda-2,12*E*-dien-14-oic acid (49)	Cytotoxicity [91]	37.35–39.31 μM	HeLa, MCF-7, and A549
	16,16-dimethoxy-cleroda-3,13*Z*-dien-15-oic acid (50)	Cytotoxicity [32]	22.43 μM	SMMC-7721
	Polylauiester A (51)	Cytotoxicity [91]	33.21–35.65 μM	HeLa, MCF-7, and A549
	Polylauiamide B (52)	Cytotoxicity [91]	28.09–29.25 μM	Same as above
	Polylauiamide C (53)	Cytotoxicity [91]	25.01–30.30 μM	Same as above
	Polylauiamide D (54)	Cytotoxicity [91]	26.73–28.88 μM	Same as above
	solidagonal acid (55)	Cytotoxicity [27]	14.67–18.12 μg/mL	MCF-7 and MDA-MB-231
		Anti-bacterial [85]		Gram-positive bacteria
			31.25 μg/mL	*B. subtilis*, *C. hoffmanii*, and *S. saprophyticus*
	ENT-kaur-16-en-19-oic acid (56)	Anti-viral [92]	13.7 μg/mL	Anti-syncytium assay against HIV
**Triterpene**	Suberosol (57)	Anti-viral [23]	3 μg/mL	Inhibition of HIV replication in H9 lymphocytes
		Cytotoxicity [93]	34.30 μg/mL	SPC-A-1
			15.02 μg/mL	SGC-7901
	24-Methylenecycloartane-3β, 16β, 23β-triol (longitriol) (58)	Cytotoxicity [83]	10.03–30.88 μg/mL	KB, MCF-7, A549, C33A
		Cytotoxicity [94]	19.3–23 μM	MDA-MB-231 and SF-268
			40.3 μM	MRC-5
		Apoptosis [94]	40 μM	NCI-H460
**Triterpenoids**	Friedelin (59)	Anti-bacterial [95]	5 μg/mL	*E. coli* and *M. tetragenus*
	Stigmast-4-ene-6α-ol-3-one (60)	Anti-bacterial [95]	5 μg/mL	Same as above
**Flavonoids**	Quercetin (61)	Anti-oxidant [54]	1.56 μg/mL	Trolox equivalent antioxidant capacity (TEAC) assay
	Quercetin-3-O-β-glucopyranoside (62)	Anti-oxidant [54]	1.56 μg/mL	TEAC assay
	Rutin (63)	Anti-oxidant [54]	1.56 μg/mL	TEAC assay
**Others**	Crassalactones A (64)	Cytotoxicity [96]	0.18–1.9 μg/mL	P-388, KB, Col-2, BCA-1, Lu-1, and ASK
	Crassalactone B (65)	Cytotoxicity [96]	3.8 μg/mL	P-388
	Crassalactone D (66)	Cytotoxicity [96]	1.1–4 μg/mL	P-388, KB, Col-2, BCA-1, and ASK
	Aristolactam AII (67)	Cytotoxicity [96]	2.7 μg/mL	P-388
	(+)-Tricinnamate (68)	Cytotoxicity [96]	3.1 μg/mL	P-388
	(+)-Rumphiin (69)	Cytotoxicity [39]	63.2–187.6 μg/mL	SPC-A-1 and K562
	α-Spinasterol (70)	Cytotoxicity [97]	60.07 ± 7.10 nM/ml	Caco-2
	Dehydrogoniothalamin (71)	Anti-inflammatory [65]	11.6 ± 1.2 μM	Inhibition of fMLP/CB-induced superoxide anion production by human neutrophils
			6.8 ± 0.9 μM	inhibition of elastase release by human neutrophils
	Goniothalamin (72)	Anti-inflammatory [65]	8.3 ± 1.4 μM	Inhibition of fMLP/CB-induced superoxide anion production by human neutrophils
			15.4 ± 1.1 μM	inhibition of elastase release by human neutrophils
	(−)-5-Hydroxy-goniothalamin (71)	Cytotoxicity [65]	7.9 μM	A549
		Anti-inflammatory [65]	8.1 ± 2.3 μM	Inhibition of fMLP/CB-induced superoxide anion production by human neutrophils
			14.6 ± 0.7 μM	Inhibition of elastase release by human neutrophils
	Octadeca-9,11,13-triynoic acid (72)	Anti-bacterial [19]	6.25 μg/mL	*M. tuberculosis*
		Cytotoxicity [19]	13 μg/mL	BC1
	α-Humulene (73)	Anti-bacterial [19]	6.25 μg/mL	*M. tuberculosis*
	F2 peptide	Apoptosis [98]	30 μg/mL	A549 and HeLa

Index of cell lines. Lung cancer: A549, GLC4, NCI-H460, NCI-H187, Lu-1, and SPCA-1. Gastric cancer: AGS, SGC-7409, and SGC-7901. Liver cancer: HA59T, HepG, Hep3B, BEL-7402, HCC-S102, and SMMC-7721. Breast cancer: BC1, BCA-1, MCF-7, MDA-MB-231, and ZR75-1. Uterine and cervix cancer: C33A and HeLa. Skin cancer: Mel2 and A431. Blood cancer: HL-60, K562, MOLT-4, and U937. Lympoma: P-388 and HT. Head and neck cancer: KB. Urothelial cancer: 786-O, A498, and T24. Prostate cancer: LNCaP. Brain cancer: ASK, SF-268, GBM8401, C6, and N18. Colon cancer: Col-2, HT-29, SW-480, Caco-2, DLD1, and SW620. Normal immortalized cell lines: NIH3T3, RAW264.7, Vero, and MRC-5.

**Table 2:** Anti-tumor effects of 16-hydroxycleroda, 3,13-dien, and 15,16 olide on different cancer cell lines.

Cell Type	Effective Dose	Effects	Effects on Signaling Pathway	Effects on Cell Cycle	References
**Leukemia**					
HL-60	10–30 μM	Apoptosis	Aurora B ↓		[45]
K562	10–30 μM	Apoptosis	Caspase-3 and -9 cleavage ↑ Aurora B, pPI3K, pAkt ↓ pJNK ↑ Survival signaling: FoxO3, FoxO4 ↑ Cell-cycle related proteins: p21 ↑ Cyclin A, cyclin B, CDK1, CDK2 ↓	G2/M phase arrest	[99]
			PRC2 complex: EZH2, Suz12 ↓		
**Head and neck cancer**					
OECM1, SAS	10–50 μM	Autophagy	LC3-II and beclin-1 ↑ Cyclin D1 ↓	G0/G1 phase arrest (SAS cells)	[36]
**Glioma**					
N18, C6	3–10 μM	Autophagy	p-p38 MAPK, pERK1/2↑	G0/G1 phase arrest	[34]
		Apoptosis	Bad, Bax, and p53 ↑ ROS overproduction Inhibition of SOD, GSH, GST, GPx activities		
**Colon cancer**		Anti-migration	Rac1, cdc42, pFAK, and FAK ↓		[53]
Caco-2	2.30 μM (48 h)	Apoptosis	cleavage of caspase-3, -8, and -9 ↑ Inhibition of growth factor-related signalling: Akt, PCNA ↓ cell cycle related proteins: p21 and p53 ↑ Inflammatory signalling: COX2, NF-κB ↓	G2/M phase arrest	[72]
**RCC**	10–40 μM	Anti-migration Anti-invasion anoikis	pFAK, FAK, pSrc, paxillin ↓, vimentin, vinculin, pNF-kB ↓, MMP2, MMP9, VEGF ↓	G2/M phase arrest	[100]
786-O, A-498					
	10–40 μM	Apoptosis	pMEK1/2, pERK1/2, pAkt, pmTOR ↓ ROS overproduction, Cytochrome c release Caspase-3, PARP-1 cleavage ↑ cMyc, HIF-2α, HSP70, Bcl-2 ↓ Cyclin B1, cyclin D1, cyclin E, CDK1↓, CDK2, CDK4 ↓ FoxO3a, p21, p53 ↑		[44]
Bladder cancer	10–40 μM	Apoptosis	cyclin D1, CDK2 and CDK4 ↓ Increase of p21, p27Kip1 and p53 ↑ Caspase-3, PARP-1 cleavage, pH2A.X ↑ Cytochrome c release, ROS overproduction, Bcl-2↓ pEGFR, pMEK, pERK1/2, pAkt↓, pmTOR, p-P70S6K ↓ HIF-1α, cMyc, VEGF ↓	G0/G1 phase arrest	[52]
T24					

## Data Availability

Not applicable.

## Abbreviation

CD: 16-hydroxycleroda-3,13-dien-16,15-olide.
